# Divalent metal transporter-related protein restricts animals to marine habitats

**DOI:** 10.1038/s42003-021-01984-8

**Published:** 2021-04-12

**Authors:** Mieko Sassa, Toshiyuki Takagi, Azusa Kinjo, Yuki Yoshioka, Yuna Zayasu, Chuya Shinzato, Shinji Kanda, Naoko Murakami-Sugihara, Kotaro Shirai, Koji Inoue

**Affiliations:** 1 Graduate School of Frontier Sciences, The University of Tokyo, Kashiwa-shi, Chiba Japan; 2 Atmosphere and Ocean Research Institute, The University of Tokyo, Kashiwa-shi, Chiba Japan; 3 Marine Genomics Unit, Okinawa Institute of Science and Technology Graduate University, Kunigami-gun, Okinawa Japan

**Keywords:** Molecular evolution, Animal physiology

## Abstract

Utilization and regulation of metals from seawater by marine organisms are important physiological processes. To better understand metal regulation, we searched the crown-of-thorns starfish genome for the divalent metal transporter (DMT) gene, a membrane protein responsible for uptake of divalent cations. We found two DMT-like sequences. One is an ortholog of vertebrate DMT, but the other is an unknown protein, which we named DMT-related protein (DMTRP). Functional analysis using a yeast expression system demonstrated that DMT transports various metals, like known DMTs, but DMTRP does not. In contrast, DMTRP reduced the intracellular concentration of some metals, especially zinc, suggesting its involvement in negative regulation of metal uptake. Phylogenetic distribution of the DMTRP gene in various metazoans, including sponges, protostomes, and deuterostomes, indicates that it originated early in metazoan evolution. However, the DMTRP gene is only retained in marine species, and its loss seems to have occurred independently in ecdysozoan and vertebrate lineages from which major freshwater and land animals appeared. DMTRP may be an evolutionary and ecological limitation, restricting organisms that possess it to marine habitats, whereas its loss may have allowed other organisms to invade freshwater and terrestrial habitats.

## Abstract (web summary)

Mieko Sassa et al. report a novel divalent metal transporter protein (DMTRP) in the crown-of-thorns starfish genome and determine that all organisms with a DMTRP gene are located in marine habitats. They also show in a functional yeast system that DMTRP can prevent uptake of certain metals, bringing insight into the evolution of metal regulation for marine organisms.

## Introduction

Seawater contains various metal ions^1^. Marine organisms utilize, and also are greatly influenced by metals in seawater^2^. For example, Zn and Cu are trace essential elements involved in various physiological processes, while the biological roles of some other elements, such as Cd and Pb, are not well known^3–5^. Especially, Zn is essential for various protein functions, and is considered to have influenced eukaryote evolution^5^. However, even trace essential elements become harmful if their concentrations exceed certain levels^6,7^; thus, metal regulation is essential for life in the sea. In addition, metal regulation systems seem to have diversified in marine organisms. For example, metals generally known as non-essential trace elements are detected in the bodies of certain marine fish and invertebrates^8^, and some marine bacteria can use cadmium as a substitute for zinc^9^. To understand metal regulation, in this study, we focused on the divalent metal transporter (DMT), which is also called natural resistance-associated macrophage protein 2 (Nramp2), a member of the SLC11 family, comprising proton-coupled metal ion transporters^10,11^. DMT has been identified in many species from bacteria to humans^12^, and transports a wide variety of metal ions. For example, rat DMT, expressed in *Xenopus* oocytes, transports Fe^2+^, Cd^2+^, Co^2+^, Cu^2+^, Ni^2+^, Mn^2+^, Pb^2+^, and Zn^2+^ ^13^. Uptake of radioisotopes of Fe^2+^, Mn^2+^, Co^2+^, Zn^2+^, and Cd^2+^ by DMTs of mammals and yeast has been documented using *Xenopus* oocytes^14–16^. The crystal structure of DMT has been determined^17^, and it has been reported that the amino acid residues DPGN and MPH, located in TM1 and TM6, are particularly important for uptake of metals^18^.

Other members of the SLC11 family include Nramp1, which regulates macrophage activation against infectious and autoimmune diseases^19^, but it occurs only in mammals. Teleost fishes lack Nramp1, but an additional family of Nramp genes that appeared during a third round whole genome duplication (3R) has been discovered and it serves Nramp1-like functions^20^. In plants, many more paralogs have evolved subfunctionalized roles, e.g., transport of specific metals or different tissue distributions^21,22^. Thus, DMTs have diversified into various lineages to accommodate specific physiological demands. However, there are few reports about DMTs of marine invertebrates, despite requirements imposed by marine environments^23^.

In this study, we identified DMT in the crown-of-thorns starfish (COTS) *Acanthaster planci*, for which a well-assembled genome and exhaustive transcriptomic information are available^24^. Based on the sequences detected, we cloned two different cDNAs encoding DMT-like proteins, one of which is an ortholog of known DMTs, but the other encodes an unknown membrane protein specific to marine invertebrates. We further analyzed their functions using a yeast expression system, and found that the unknown protein is quite unlike known DMTs. Finally, we will discuss the possible significance of this protein gene relative to the habitat transition of animals from the sea to freshwater and land.

## Results

### Two distinct DMT-like proteins in COTS

**Fig. 1:**
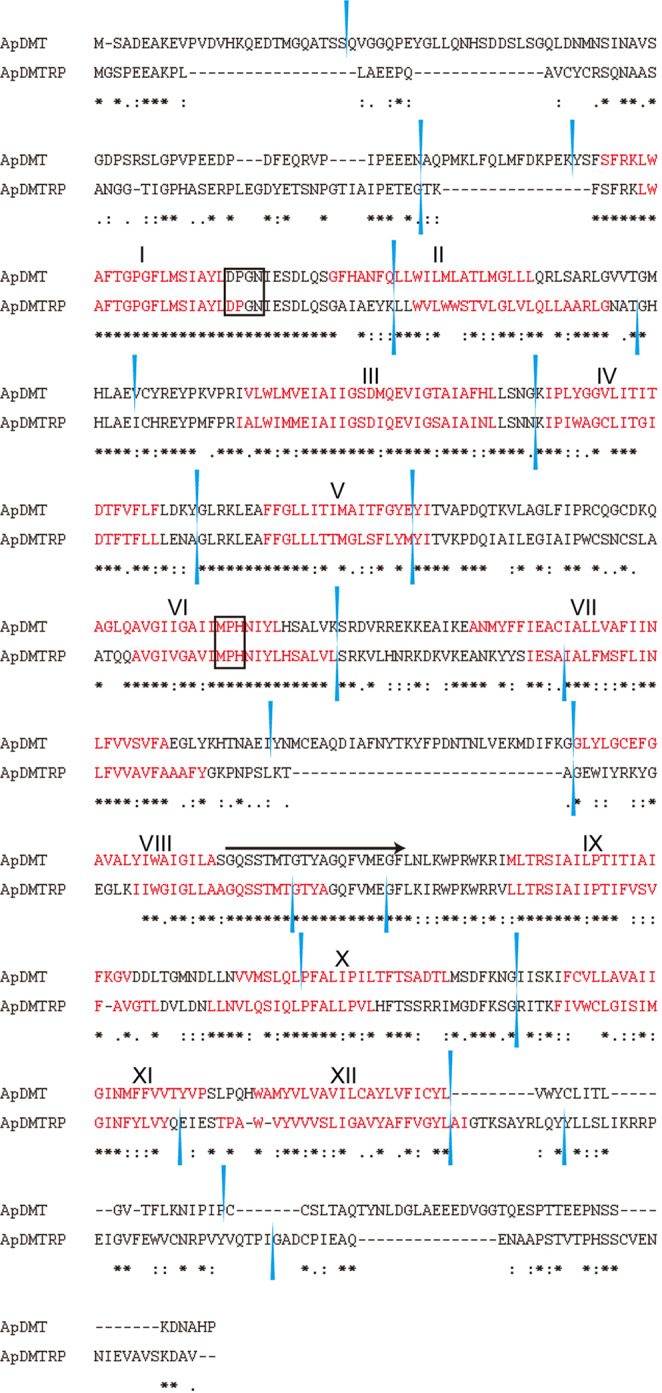
Comparison of amino acid sequences of the crown-of-thorns starfish (COTS), *Acanthaster planci*, divalent metal transporter (ApDMT) and DMT-related protein (ApDMTRP). Alignment was carried out with MAFFT version 7. Identical amino acids are indicated by asterisks. Conservative and semi-conservative substitutions are indicated with colons and periods, respectively. Black arrows indicate consensus transport motifs (CTMs). Blue arrowheads indicate positions of introns. Black boxes indicate functionally important amino acid residues.

**Fig. 2:**
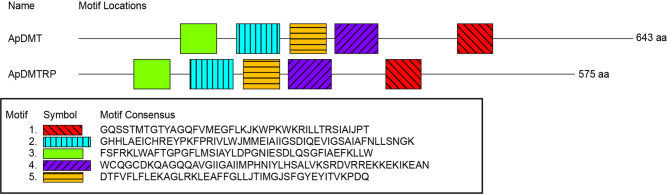
Conserved motif locations of the crown-of-thorns starfish (COTS), *Acanthaster planci*, divalent metal transporter (ApDMT) and DMT-related protein (ApDMTRP). Conserved domains were identified using the MEME web server. Colored boxes indicate motifs. Black lines indicate whole proteins and numbers on the right side indicate the length of the encoded amino acid sequence of each gene.

Using the scallop DMT, the only marine invertebrate DMT whose multiple metal ion transport activities have been demonstrated electrophysiologically^23^, as a query, a database search of the COTS genome detected two genes encoding DMT-like sequences. They are named DMT and DMTRP, based on results of the molecular phylogenetic analysis below. Alignment results of the scallop DMT with ApDMT and ApDMTRP are shown in Fig. S1. cDNAs of the two genes were cloned from starfish stomach because the fragments per kilobase per million reads mapped (FPKM) obtained from the database https://marinegenomics.oist.jp/cots/blast/search?project_id=46 (Fig. S2) suggested that the stomach is the site of highest expression for both genes, which display 52.76% identity. By aligning cDNA and genome sequences, 16 and 17 exons were identified in the DMT and DMTRP genes, respectively (Fig. 1). Intron positions are conserved between the two sequences at nine locations. Both are estimated to have 12 transmembrane domains, manifesting conserved locations. Consensus transport motifs (CTM)^12,25,26^ are also highly conserved. In addition, the sequences “DPGN” in transmembrane domain (TM) 1 and “MPH” in TM6 (Fig. 1), both of which have been reported as functionally important, are also conserved^18^. By motif search, common domains were identified in the same order (Fig. 2). Such common structural characteristics suggest that both genes were derived from a common ancestral gene.

**Fig. 3:**
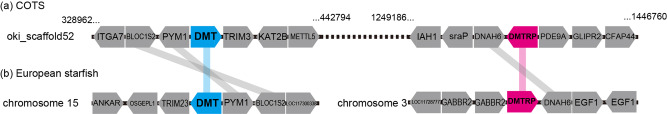
Genes around divalent metal transporter (DMT) and DMT-related protein (DMTRP) genes. **a** The crown-of-thorns starfish (COTS), *Acanthaster planci* and **b** the European starfish, *Asterias rubens*. Scaffold names and chromosome numbers are indicated on the left. Genes and their orientations are indicated by pentagons of the same size. DMT, DMTRP, and other genes are indicated in blue, magenta, and gray. Numbers above the COTS scaffold indicate start or end positions of gene locations. Protein names and gene IDs are listed in Table S2.

Both genes occur on the same scaffold; however, they are separated by about 900 kb, and no syntenic genes were found between them (Fig. 3). Since we were able to detect DMT and DMTRP genes in the genome database of the European starfish (*Asterias rubens*), we performed a synteny comparison the same way. The DMT gene was found on chromosome 15, and the DMTRP gene was on chromosome 3, and they each share one common neighboring gene with their COTS orthologs. However, no common syntenic genes were found between the DMT and DMTRP genes (Fig. 3). Thus, the evolutionary relationship of the DMT and DMTRP cannot be inferred by gene synteny. In addition, no common syntenic gene was found around DMT and DMTRP genes between COTS and the sea urchin, *Strongylocentrotus purpuratus* (Fig. S3), suggesting low conservation of gene synteny around the DMT and DMTRP genes.

### Phylogenetic relationship of the two DMT-like proteins

As a result of a BLASTP search using the cloned sequences as queries, DMT-like sequences of 48 organisms from bacteria to humans were obtained (Supplementary Data 1). A phylogenetic analysis was performed on those obtained sequences using the maximum-likelihood (ML) method. In the resulting phylogenetic tree (Fig. S4), bacterial sequences formed a single clade. Plant-derived and amoebozoa-derived sequences formed two separate clades, one of which occupies a position close to bacteria, suggesting its relationship with bacterial sequences. Especially, *Dictyostelium discoideum* AX4 1 may have resulted from horizontal gene transfer^27^ because it lacks introns, whereas another gene, *D. discoideum* AX4 2, has an intron (Gene ID: 8619995). In contrast, all metazoan sequences formed a single clade (Fig. S4) at a position close to the Choanoflagellate sequence (the organism most closely related to metazoans). Although the position of the metazoan DMT clades is also close to one of the plant DMT clades, the order of diversification is still unpredictable because it is difficult to root this tree.

**Fig. 4:**
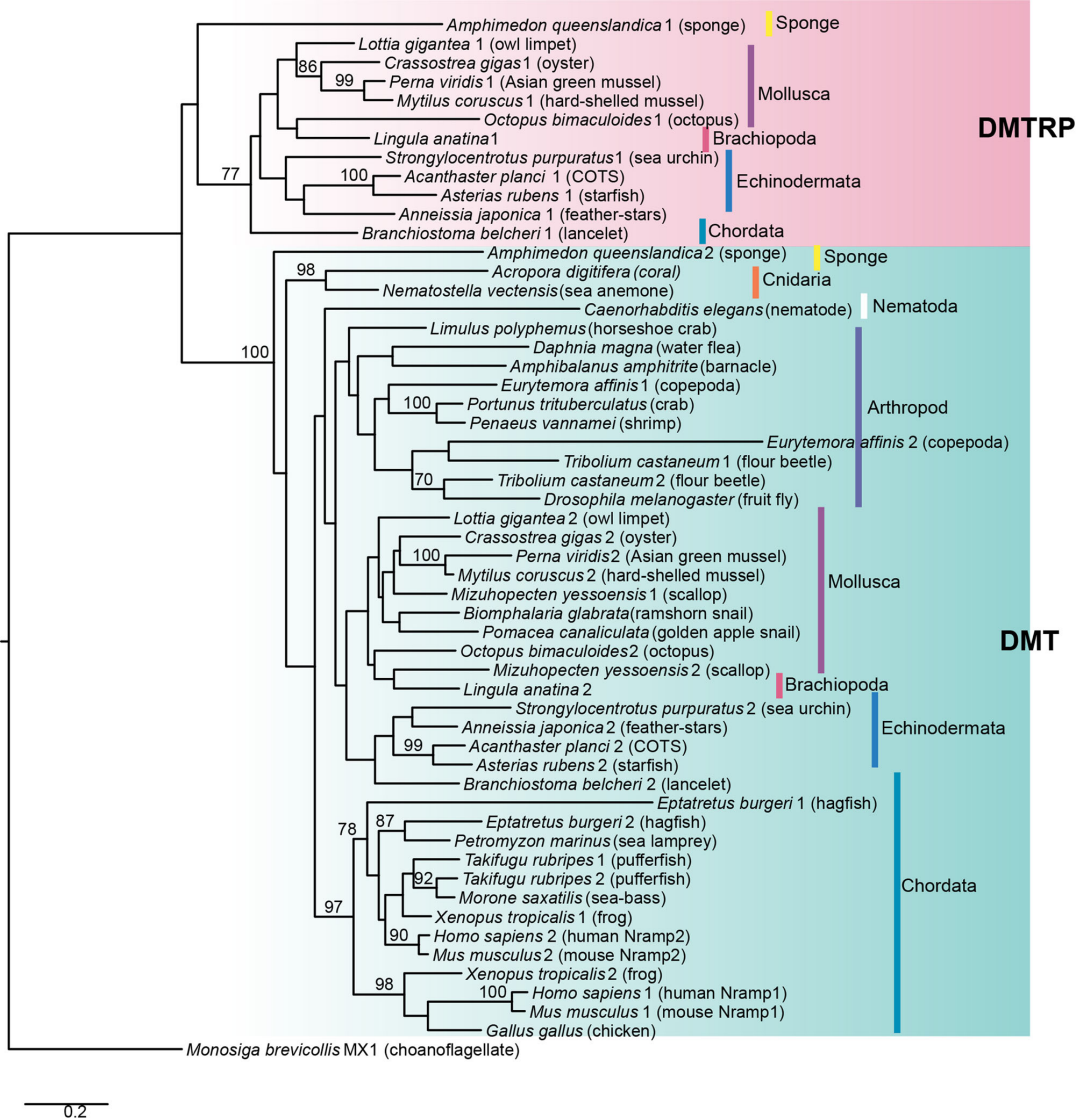
Maximum-likelihood trees of divalent metal transporter (DMT)-like protein sequences obtained from databases. A tree constructed using metazoan sequences. The tree was rooted using the sequence of a choanoflagellate, *Monosiga brevicollis*, as an outgroup. Bootstrap values more than 70% are indicated at nodes. The scale bar represents a phylogenetic distance of 0.2 substitutions per site.

Branching patterns within the metazoan clade of this tree were not robust, possibly due to limited alignment lengths, since the tree included bacterial, fungal, and plant sequences. Therefore, we conducted another molecular phylogenetic analysis using 32 metazoan sequences with Choanoflagellates as an outgroup (Fig. 4). As a result, metazoan sequences divided into two clades, each of which contained one of the COTS DMT-like sequences. Thus, we found that two DMT-like clades diverged after the divergence of the Metazoa, but before the differentiation of sponges.

All known DMTs reside in one clade, confirming that the COTS sequence belonging to this clade is homologous. We named it ApDMT (*A. planci* DMT). The other clade contained DMTRP sequences that have not been reported previously. In tetrapods, two known DMTs, Nramp1 and Nramp2, belonging to the DMT clade, suggesting that their divergence occurred in the vertebrate lineage, as reported previously^20^. However, DMTRP is distinct from Nramp1.

Both clades included protostome and deuterostome sequences. Moreover, sponge sequences also occurred in both clades, indicating the divergence of DMT and DMTRP in an early ancestor of metazoans. Phylogenetic relationships of sequences in the DMT and DMTRP clades conflict with presumed phylogeny of many species, suggesting that DMT and DMTRP sequences have diversified in each taxon. Interestingly, all organisms possessing a DMTRP gene, including sponges, molluscs, echinoderms, and lancelets, possess the DMT gene as well. Moreover, all DMTRP-bearing animals are marine species. The DMTRP gene was not detected in vertebrates, insects, or nematodes, which are among the most successful freshwater and terrestrial animal taxa. Importantly, DMTRP does not occur in two freshwater gastropods either, the ramshorn and golden apple snails, although it is present in a marine gastropod, the owl limpet.

### Functional analyses

**Fig. 5:**
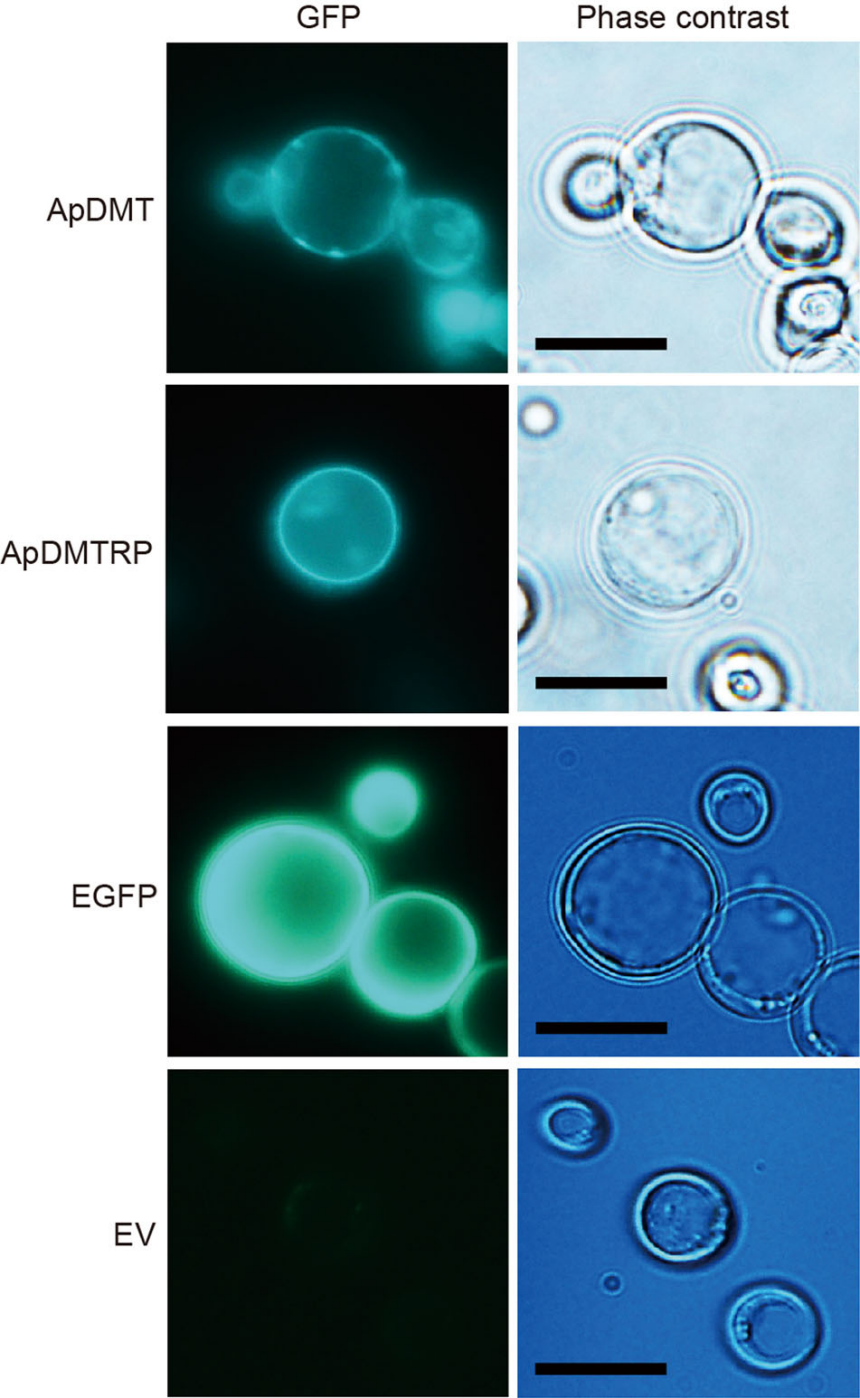
Subcellular localization of the crown-of-thorns starfish (COTS), *Acanthaster planci*, divalent metal transporter (ApDMT) and DMT-related proteins (ApDMTRP) fused with EGFP expressed in yeasts. Yeasts (DY1457) were transformed with ApDMT and ApDMTRP genes fused to the EGFP gene and observed with fluorescent and phase contrast microscopy. Yeasts expressing EGFP alone (EGFP) and those transformed with an empty vector (EV) are shown for comparison. The scale bar is 10 μm.

Functional differences between ApDMT and ApDMTRP were examined using a yeast (*Saccharomyces cerevisiae*) expression system. First, localization of expression products on the yeast membrane was confirmed by adding the enhanced green fluorescent protein (EGFP) gene downstream of the DMT and DMTRP genes. As the result, fluorescence of ApDMT-EGFP and ApDMTRP-EGFP fusion proteins was observed on the cell membrane as expected (Fig. 5).

**Fig. 6:**
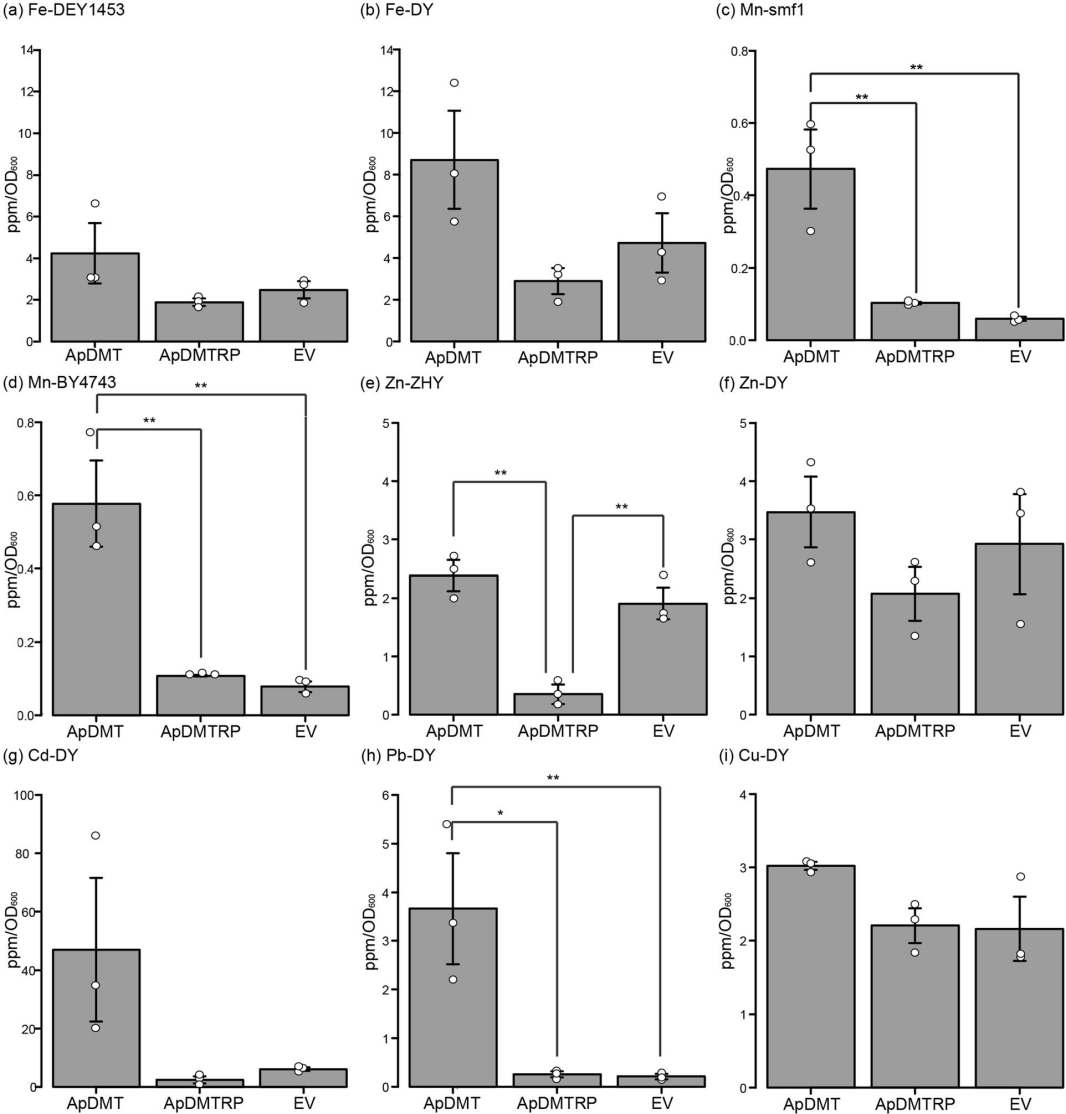
Heavy metal accumulation in yeasts in which the crown-of-thorns starfish (COTS), *Acanthaster planci*, divalent metal transporter (ApDMT) and DMT-related proteins (ApDMTRP) were expressed. Wild-type or metal uptake-deficient strains of yeasts were transformed with the expression vector pDR195 containing ApDMT or ApDMTRP or empty vector (EV), and exposed to media containing FeCl_3_ (**a**, **b**), MnSO_4_ (**c**, **d**), ZnCl_2_ (**e**, **f**), CdCl_2_ (**g**), PbCl_2_ (**h**), or CuCl_2_ (**i**). The vertical axis shows the intracellular concentration measured by ICP-MS normalized by yeast optical density (OD_600_). The horizontal axis shows the names of expressed genes/yeast strains. Each bar represents the mean ± SEM (*n* = 3). One or two asterisks on the bar represent statistical significance by one way ANOVA with Tukey’s post-hoc HSD test at *p* < 0.01(**) or *p* < 0.05(*). DY, wild-type strain of DEY1453 and ZHY3; DEY, Fe-uptake-deficient yeast strain DEY1453; ZHY, Zn-uptake-deficient yeast strain ZHY3; BY4743, wild-type strain of single mutant strain HomDip-YOL122C lacking SMF1; smf1, Mn-uptake-deficient yeast strain HomDip-YOL122C lacking SMF1. Dot plots shows individual data points. Dot plots data are in Supplementary Data 2.

Subsequently, ApDMT and ApDMTRP, without EGFP, were expressed in yeast, and the yeast was exposed in separate experiments to one of six metals (Fe, Mn, Zn, Cd, Cu, and Pb). After exposure, metals in the cells were quantified using inductively coupled plasma-mass spectrometry (ICP-MS). For Fe, Mn, and Zn exposure tests, Fe^2+^, Zn^2+^, or Mn^2+^ uptake-deficient mutants lacking specific metal transporter genes^28–30^ were used in addition to wild-type strains. When exposed to Fe, ApDMT-expressing mutant and wild-type strains tended to accumulate more Fe than the control, while Fe accumulation tended to decrease in ApDMTRP-expressing yeast. The level of Fe in DMT-expressing yeast was significantly higher than DMTRP-expressing yeast when the wild-type strain was used (Fig. 6a, b).

In the case of Mn exposure, Mn level was significantly higher in ApDMT-expressing yeast than in control or ApDMTRP-expressing yeast, in both mutant and wild-type strains (Fig. 6c, d). In the case of Zn exposure, the ApDMTRP-expressing yeast accumulated Zn significantly less than the control and ApDMT-expressing yeast (Fig. 6e). A similar trend that ApDMTRP-expressing yeast accumulate Zn less than the control and ApDMT-expressing yeast was observed in an experiment using wild-type strains, although it was not statistically significant (Fig. 6f). For Cd, Pb, and Cu, for which no uptake-deficient mutants are available, experiments were conducted only in wild-type strains. ApDMT-expressing yeast tended to accumulate Cd compared to the control and ApDMTRP-expressing yeast, while ApDMTRP-expressing yeasts tended to decrease Cd levels compared to the control (Fig. 6g). ApDMT-expressing yeast significantly increased Pb accumulation relative to the control (Fig. 6h). The intracellular concentration of Cu was higher in ApDMT-expressing yeast than in ApDMTRP-expressed yeast and controls, but the difference was statistically insignificant (Fig. 6i).

## Discussion

In this study, we discovered two genes encoding DMT-like sequences in the COTS genome. By phylogenetic analysis, one was identified as an ortholog of known DMTs from other organisms. The other gene encoded a unknown protein that we named DMTRP. Both genes were similar in sequence, arrangement of transmembrane domains and functional motifs, and positions of introns, suggesting that they originated from the same ancestral gene.

Molecular phylogenetic analysis showed that both DMT and DMTRP clades contained sequences from sponges, protostomes, and deuterostomes. However, only a single DMT-like gene was found in a choanoflagellate, *Monosiga brevicollis*, a unicellular organism most closely related to metazoans^31^. Thus, the divergence of DMT and DMTRP is likely to have occurred at an early stage of metazoan evolution. We also attempted to characterize the origin of DMTRP based upon gene synteny. The DMT and DMTRP genes were detected on the same scaffold in COTS, but their positions were 900 kb apart and no common syntenic genes were found around them. In the European starfish, DMT and DMTRP genes are on separate chromosomes (Fig. 3). In addition, DMT and DMTRP genes of oyster (*Crassostrea gigas*) are also localized on different chromosomes (Fig. S3). It seems that the localization of the two genes on the same scaffold in COTS may be the result of chromosome fusion or incidental translocation to the same chromosome that occurred specifically in COTS. Moreover, shared synteny between the regions containing the DMT and DMTRP genes does not exist in the European starfish genome. Therefore, the divergence of DMT and DMTRP could not be inferred from synteny. However, these results are consistent with the result of phylogenetic analysis indicating their ancient divergence.

As mentioned above, the structures of ApDMT and ApDMTRP are very similar. Amino acid residues that are essential for DMT function are well conserved in ApDMTRP (Fig. 1)^17,18,32,33^. Thus, as an original working hypothesis, we expected that the functions of ApDMT and ApDMTRP would be similar. In addition, FPKM data (Fig. S2) indicated that the stomach was the site of highest expression for both the ApDMT and ApDMTRP genes, suggesting that both are involved in regulating metal uptake in the digestive system. However, functional analysis by the expression system in yeast revealed that ApDMT and ApDMTRP have different functions. Fe, Cd, Zn, Mn, Pb, and Cu, tended to increase in yeasts expressing ApDMT, suggesting that it transports these metals into cells, as has been reported for mammalian DMTs^13^. In contrast, expression of ApDMTRP did not increase intracellular metal levels, other than Mn. In fact, the intracellular concentration of Zn in ApDMTRP-expressing yeast was significantly lower than that of a Zn-uptake-deficient strain. A similar result was obtained with wild-type yeast, even though the difference was not statistically significant. A similar trend was also observed for Fe and Cd. These results suggest that metal uptake is not ApDMTRP’s primary function, but that it reduces metal levels, especially Zn, although the mechanism employed remains to be discovered. That DMTRP does not import metals is also consistent with the fact that all species that have DMTRP also have DMT.

Interestingly, only marine organisms possess DMTRP. Despite the many animal genomes now available, the DMTRP gene does not appear in the genome of any freshwater aquatic or terrestrial animal. Therefore, DMTRP must have a role specific to marine animals. The most remarkable function of the DMTRP demonstrated in this study is the prevention of Zn uptake (Fig. 6). Even though Zn is an essential metal for some physiological functions, elevated cellular concentrations of Zn are toxic^34^; thus, marine organisms must maintain tissue Zn levels within specific ranges^35^. The vertical distribution of both Fe and Zn increases with ocean depth, but at most sites, Zn concentrations are higher than Fe, by up to 10-fold^36^. In addition, Zn levels in the ocean have been maintained at the levels comparable to the present since the occurrence of ancestral eukaryotes or before that^5^. Therefore, it seems reasonable that DMTRP regulates concentrations of specific metals, especially Zn, by exporting or preventing uptake of those that occur naturally in concentrations harmful to the organisms. In contrast, retention of DMTRP may be disadvantageous for freshwater and terrestrial environments where Zn is less abundant^35^.

From an evolutionary viewpoint, our molecular phylogenetic tree (Fig. 4) showed that the disappearance of the DMTRP gene from vertebrate and insect lineages involves multiple, separate events. The DMTRP gene is detected in lancelets, but not in hagfish, which are obligate marine organisms^37^, so it may be that the loss of DMTRP occurred in an ancestral vertebrate before the transition into freshwater. DMTRP is also absent in marine crustaceans and insects, suggesting that its loss is common among arthropods. Furthermore, DMTRP is not detected in the nematode, *Caenorhabditis elegans*, suggesting that DMTRP loss occurred much earlier, in an ancestor of ecdysozoans. However, nematodes, crustaceans, and fish are abundant in the sea. Thus, they must have a function that substitutes for that of DMTRP. Although such substitutive mechanism is unknown at present, it may be related to metallothionein, which also binds these metals in the cell and reduces their concentrations to tolerable levels^38^. Interestingly, taxa in which metallothioneins have been discovered, e.g., Nematoda, Arthropod, Chordata, and Cnidaria, approximately match those that have not retained DMTRP^39^. Regardless, results of this study suggest that freshwater and terrestrial animals are descended only from lineages that lack DMTRP. DMTRP seems to be an evolutionary and ecological roadblock, which restrict animals to marine environments.

## Methods

### Starfish sample collection and cDNA synthesis

A live COTS, *A. planci*, was collected in Okinawa, Japan. Since DMT is highly expressed in the stomach, that organ was dissected and quickly frozen in liquid nitrogen and stored at −80 °C until use. Total RNA was extracted from the intestine and stomach using TRIsure (BIOLINE, London, UK). DNase treatment employed Deoxyribonuclease I, Amplification Grade (Invitrogen, Carlsbad, CA). A double-stranded cDNA pool was synthesized from 455 ng total RNA with a SuperScript III First-Strand Synthesis System for RT-PCR (Invitrogen) and used as a cDNA template for PCR.

### cDNA cloning

Throughout this study, KOD Plus DNA polymerase (TOYOBO, Osaka, Japan) was used for PCR and reaction mixtures were prepared according to the manufacturer’s protocol. Primers for PCR are listed in Table S1. Thermal cycler conditions were as follows: initial denaturation at 94 °C for 2 min, followed by 35 cycles of 94 °C for 15 s, 60 °C for 30 s, 68 °C for 2 min 30 s and a final extension at 72 °C for 7 min. In cases in which the amplified product was to be subcloned into T-vector, 5 μL Ex Taq (Takara Bio, Kusatsu, Japan) were added before the last cycle. This procedure was also used for other PCR experiments unless otherwise indicated. ApDMT cDNA was amplified using the primers ApDMT-5UTR and ApDMT-3UTR. Similarly, ApDMTRP cDNA was amplified using the primers ApRP-5UTR and ApRP-3UTR. Sequences of these amplicons were determined using an ABI PRISM 3130xl Genetic Analyzer (Applied Biosystems, Waltham, MA, USA).

### Phylogenetic analyses

Using ApDMT and ApDMTRP sequences as queries, similar sequences were collected from the literature or by BLASTP searches of whole genome databases using non-redundant protein sequences (nr) at NCBI. In addition, hagfish sequences were derived from Ensembl [https://www.ensembl.org/Eptatretus_burgeri/Info/Index?db=core]. Sequences of *Perna viridis* were identified from the genomic sequence data^40^, and Nramp1, a member of the Nramp family, was obtained from the NCBI database by searching by name. Similarity searches employed a cutoff of 1.0E^−100^, and sequences that were considered transcript variants by phylogenetic analyses were excluded. Detected sequences were aligned using MAFFT version 7^41,42^, and trimmed automatically with trimAL v1.2 [http://trimal.cgenomics.org/]^43^ with the strictplus program. A ML tree was constructed using version 7.2.6 of RAxML [http://wwwkramer.in.tum.de/exelixis/software.html], with PROTCATWAG model^44^, and bootstrap replications were set to 1000. An ML tree containing 35 metazoans was also constructed using the same procedure, setting the choanoflagellate, *M. brevicollis*, as an outgroup, because it is a close ancestor to metazoans.

### Domain and motif analyses

Phobius was used to predict transmembrane regions^45^. To identify conserved motifs, Multiple Em for Motif Elicitation Version 5.1.1 (MEME, http://meme-suite.org/tools/meme) was used with default parameters, except that the maximum number of motifs was 5^46^.

### Strains, media, culture conditions, and construction of expression vectors

*Saccharomyces cerevisiae* strain DY1457, and its low-affinity iron transport mutant DEY1453^28^, low-affinity zinc transport mutant ZHY3^29^, as well as strain BY4743 were provided by Dr. David J. Eide and Dr. Ihsan Ullah. The yeast expression vector pDR195 (Addgene plasmid #36028) and a single mutant strain of BY4743, HomDip-YOL122C, lacking SMF1, the yeast DMT known as “Suppressor of Mitochondria import Function”^30^ were purchased from Addgene (Cambridge, MA, USA) and Transomic Technologies (Huntsville, AL, USA), respectively. The list of yeasts used in this study is shown in Table 1. Using ApDMT and ApDMTRP as a template, the Kozak sequence was added to them by PCR with primers KozakApDMT and ApDMT3 for ApDMT and KozakApRP and Ap3RP for ApDMTRP. Thermal cycler conditions described above were used, but the annealing temperature was changed as follows: 70 °C at the first cycle and gradually decreased to 60 °C within 35 cycles (touch-down PCR). The amplified fragment was subcloned into pGEM-T as described above, and resulting plasmids were named kozak-ApDMT and kozak-ApDMTRP. From these plasmids, Kozak sequence-added ApDMT and ApDMTRP-coding sequences were amplified by using primers, pDR+ApDMT5 with pDR+ApDMT3, and pDR+ApRP5 with pDR+ApRP3, respectively, for ligation to pDR195. Primers ADH5 and pPMA3 were used to amplify the vector pDR195. These amplified fragments were ligated using an In-Fusion HD cloning kit (Takara Bio Inc.). The resulting vectors, pDR195+ApDMT and pDR195+ApDMTRP, were used for functional expression experiments in yeast. For vectors to examine subcellular localization, the EGFP gene was amplified using the primers pDR-EGFP and EGFP-ApRP from pNHK12^47^,which were provided by the National Bio-Resource Project (NBRP), Japan. This amplicon was attached to the C-terminal end of pDR195+ApDMT and pDR195+ApDMTRP using primers ApDMT-EGFP and EGFP-pDR to express fusion protein in frame, using In-Fusion HD cloning kit. The resulting vectors were named pDR195+ApDMT+EGFP and pDR195+ApDMTRP+EGFP, respectively.

**Table 1:** Yeast strains used for functional analysis.

	For Fe uptake test	For Zn uptake test	For Mn uptake test	For Cd, Cu, Pb uptake test
Wild type	DY1457	DY1457	BY4743	DY1457
Mutant	DEY1453(*fet3fet4*)	ZHY3(*zrt1zrt2*)	HomDip-YOL122C lacking SMF1	–

### Confirmation of the expression of DMT and DMTRP in yeast cells

pDR195+ApDMT+EGFP, pDR195+ApDMTRP+EGFP, and empty vector pDR195 were introduced into competent yeast cells DY1457 using a frozen-EZ Yeast transformation II kit (Zymo Research Co., CA, USA). Transformants were selected on plates containing the synthetic dropout medium with appropriate amino acids. These yeasts were incubated in 1 mL of synthetic defined (SD)-Ura medium buffered at pH 6.0 with 50 mM 2-morpholinoethanesulfonic acid at 30 °C for 24 h and collected by centrifugation for 5 min at 6000 × *g*. Afterward, cells (OD_600_ = 0.1) were washed with sterile water and cultured in 1 mL SD-Ura medium (pH 6.0) at 30 °C overnight. A fluorescent microscope (Olympus BX-53 equipped with a high-pressure mercury lamp, U-HGLGPS, and a fluorescent filter cube, U-FGFP; Olympus, Tokyo, Japan) was used to observe expressed fusion proteins, and images were captured using a digital camera DS-Ri1 (Nikon, Tokyo, Japan).

### Heavy metal accumulation

Constructed vectors ApDMT+DR195 and ApDMTRP+pDR195 were introduced into DEY1435 (*fet3fet4*), ZHY3 (*zrt1zrt2*), SMF1, DY1453, and BY4743 using a Frozen-EZ Yeast Transformation II Kit. Transformants were selected using SD-Ura medium plates (pH 4.0) supplemented with 10 μM FeCl_3_ for DEY1453; SD-Ura medium plates (pH 5.8) supplemented with 10 μM each of FeCl_3_ and ZnCl_2_ for ZHY3; SD-Ura medium plates (pH 5.2) supplemented with 100 μM MnSO_4_ for SMF1; SD-Ura medium plates for Transformed DY1453 and BY4743. These yeasts were incubated in 5 mL of liquid SD-Ura medium at 30 °C for 24 h and collected by centrifugation for 5 min at 6000 × *g*. Afterward, cells (OD_600_ = 0.1) were washed with sterile water and cultured in 10 mL of liquid SD-Ura medium containing 2 µM of one of the following metal salts, FeCl_3_, ZnCl_2_, MnSO_4_, CdCl_2_, PbCl_2_, and CuCl_2_ at 30 °C overnight for 24 h at 30 °C, with shaking at 250 rpm. After that, the yeast cells were pelleted by centrifugation at 3500 rpm for 5 min, washed with ice-cold 20 mM EDTA and rinsed three times with sterile water. Pellets were dried at 70 °C for 36 h. Dried cells were digested for 48 h in 0.5 mL 68% ultrapure HNO_3_ (Tama Chemicals Co., Kanagawa, Japan, TAMAPURE-AA-100) at 60 °C. Digested solution was diluted to the appropriate concentration for the analysis, ~60 times by weight. All dilutions was performed gravimetrically. The Fe, Zn, Mn, Cd, Pb, and Cu concentrations in digested-diluted solution were quantified using ICP-MS (Agilent 7700x ICP-MS, Agilent Technologies Inc., Santa Clara) based on the sensitivity factor obtained from ICP Multi-Element Standards IV (Merck, Darmstadt, German). A procedure blank was also evaluated and found to be negligible. Metal accumulations in the cells were calculated and normalized by yeast optical density (OD_600_). Typical reproducibility and accuracy of NIST SRM2976 (mussel tissue) for Fe, Zn, Mn, Cd, Pb, and Cu were 9 and 145%, 5 and 135%, 3 and 129%, 3 and 123%, 4 and 105%, and 8 and 124%, respectively. Systematic overestimation of NIST SRM2976 likely due to an isobaric interference from organic sample, as well as uncertainty of certified/reference value of NIST SRM2976. Reasonably precise reproducibility of the NIST SRM2976 analysis certifies the reliability of relative sample differences.

### Statistics and reproducibility

Yeast experiments were performed in triplicate using separate colonies picked from plates. Statistical analyses of the data were performed using one way ANOVA with Tukey’s post-hoc HSD test in R (standard package) to show the significance of differences. A difference of *p* < 0.05 was considered statistically significant.

### Database search for DMT-like genes of COTS

To obtain DMT-like sequences, a BLASTP search was carried out in the Crown-of-thorns starfish (COTS) genome [https://www.ncbi.nlm.nih.gov/genome/7870?genome_assembly_id=301786] using the DMT of the scallop, *Mizuhopecten yessoensis* (GenBank: BAD99106.1) as a query^23^.

### Reporting summary

Further information on research design is available in the Nature Research Reporting Summary linked to this article.

## Supplementary information

Supplementary Information

Description of Additional Supplementary Files

Supplementary Data 1

Supplementary Data 2

Reporting Summary

## Data Availability

DMT and DMTRP sequences of COTS and Asian green mussel are deposited to DDBJ/EMBL/Genbank Database under the accession numbers LC585429, LC585430, LC585431, LC585432, respectively.

## References

[1] Takeda, S. et al. Bioavailability and biogeochemical processes of trace metals in the surface ocean. In *Western Pacific Air–Sea Interaction Study* (eds Uematsu, M. et al.) 163–176 (TERRAPUB, 2014).

[2] Moore CM (2013). Processes and patterns of oceanic nutrient limitation. Nat. Geosci..

[3] Ochiai E (1983). Copper and the biological evolution. BioSystems.

[4] Kalay M, Canli M (2000). Elimination of essential (Cu, Zn) and non-essential (Cd, Pb) metals from tissues of a freshwater fish *Tilapia zilli*. Turk. J. Zool..

[5] Scott C (2012). Bioavailability of zinc in marine systems through time. Nat. Geosci..

[6] Migliaccio O (2015). Maternal exposure to cadmium and manganese impairs reproduction and progeny fitness in the sea urchin *Paracentrotus lividus*. PLoS ONE.

[7] Migliaccio O (2014). Stress response to cadmium and manganese in *Paracentrotus lividus* developing embryos is mediated by nitric oxide. Aquat. Toxicol..

[8] Yılmaz AB (2017). Review of heavy metal accumulation on aquatic environment in Northern East Mediterranean Sea part I: some essential metals. Rev. Environ. Health.

[9] Jeffrey PD (2008). Structure and metal exchange in the cadmium carbonic anhydrase of marine diatoms. Nature.

[10] Nevo Y, Nelson N (2006). The NRAMP family of metal-ion transporters. Biochim. Biophys. Acta—Mol. Cell Res..

[11] Tandy S (2000). Nramp2 expression is associated with pH-dependent iron uptake across the apical membrane of human intestinal Caco-2 cells. J. Biol. Chem..

[12] Cellier M (1995). Nramp defines a family of membrane proteins. Proc. Natl Acad. Sci. USA.

[13] Gunshin H (1997). Cloning and characterization of a mammalian proton-coupled metal-ion transporter. Nature.

[14] Sacher A (2001). Properties of the mammalian and yeast metal-ion transporters DCT1 and Smf1p expressed in *Xenopus laevis* oocytes. J. Exp. Biol..

[15] Marciani P (2004). Modulation of DMT1 activity by redox compounds. J. Membr. Biol..

[16] Okubo M (2003). Cadmium transport by human Nramp 2 expressed in *Xenopus laevis* oocytes. Toxicol. Appl. Pharmacol..

[17] Bozzi AT (2016). Crystal Structure and Conformational change mechanism of a bacterial Nramp-family divalent metal transporter. Structure.

[18] Bozzi AT (2019). Structures in multiple conformations reveal distinct transition metal and proton pathways in an Nramp transporter. eLife.

[19] Blackwell JM (2000). Understanding the multiple functions of Nramp1. Microb. Infect..

[20] Neves JV (2011). Natural history of SLC11 genes in vertebrates: tales from the fish world. BMC Evol. Biol..

[21] Ullah I (2018). Evolution, and functional analysis of Natural Resistance-Associated Macrophage Proteins (NRAMPs) from *Theobroma cacao* and their role in cadmium accumulation. Sci. Rep..

[22] Qin L (2017). Genome-wide identification and expression analysis of NRAMP family genes in Soybean (*Glycine max* L.). Front. Plant Sci..

[23] Toyohara H (2005). Scallop DMT functions as a Ca 2 transporter. FEBS Lett..

[24] Hall MR (2017). The crown-of-thorns starfish genome as a guide for biocontrol of this coral reef pest. Nature.

[25] Vidal SM (1993). Natural resistance to infection with intracellular parasites: Isolation of a candidate for Bcg. Cell.

[26] Gruenheid S (1995). Identification and characterization of a second mouse Nramp gene. Genomics.

[27] Richer E, Courville P, Bergevin I, Cellier MFM (2003). Horizontal gene transfer of “prototype” Nramp in bacteria. J. Mol. Evol..

[28] Dix D (1994). The FET4 gene encodes the low affinity Fe(II) transport protein of *Saccharomyces cerevisiae*. J. Biol. Chem..

[29] Zhao H, Eide D (1996). The ZRT2 gene encodes the low affinity zinc transporter in *Saccharomyces cerevisiae*. J. Biol. Chem..

[30] West AH (1992). Two related genes encoding extremely hydrophobic proteins suppress a lethal mutation in the yeast mitochondrial processing enhancing protein. J. Biol. Chem..

[31] King N (2008). The genome of the choanoflagellate *Monosiga brevicollis* and the origin of metazoans. Nature.

[32] Ehrnstorfer IA (2014). Crystal structure of a SLC11 (NRAMP) transporter reveals the basis for transition-metal ion transport. Nat. Struct. Mol. Biol..

[33] Bozzi AT (2016). Conserved methionine dictates substrate preference in Nramp-family divalent metal transporters. Proc. Natl Acad. Sci. USA.

[34] Formigari A (2007). Zinc, antioxidant systems and metallothionein in metal mediated-apoptosis: biochemical and cytochemical aspects. Comp. Biochem. C.

[35] Neff, J. M. Zinc in the ocean. In *Bioaccumulation in Marine Organisms* (ed. Neff J. M.) 175–189 (Elsevier Science, 2002).

[36] Obata H (2017). Dissolved iron and zinc in Sagami Bay and the Izu-Ogasawara. Trench J. Oceanogr..

[37] Glover CN, Weinrauch AM (2019). The good, the bad and the slimy: experimental studies of hagfish digestive and nutritional physiology. J. Exp. Biol..

[38] Kimura T, Kambe T (2016). The functions of metallothionein and ZIP and ZnT transporters: an overview and perspective. Int. J. Mol. Sci..

[39] Ziller A, Fraissinet-Tachet L (2018). Metallothionein diversity and distribution in the tree of life: a multifunctional protein. Metallomics.

[40] Inoue, K. et al. Genomics and transcriptomics of the green mussel explain the durability of its byssus. *Sci. Rep.*10.1038/s41598-021-84948-6 (2021). 10.1038/s41598-021-84948-6 PMC7971044 33727571

[41] Katoh K, Standley DM (2013). MAFFT multiple sequence alignment software version 7: improvements in performance and usability. Mol. Biol. Evol..

[42] Katoh K (2019). MAFFT online service: multiple sequence alignment, interactive sequence choice and visualization. Brief. Bioinform..

[43] Capella-Gutiérrez S (2009). trimAl: a tool for automated alignment trimming in large-scale phylogenetic analyses. Bioinformatics.

[44] Stamatakis A (2006). RAxML-VI-HPC: maximum likelihood-based phylogenetic analyses with thousands of taxa and mixed models. Bioinformatics.

[45] Käll L (2004). A combined transmembrane topology and signal peptide prediction method. J. Mol. Biol..

[46] Bailey TL, Elkan C (1994). Fitting a mixture model by expectation maximization to discover motifs in biopolymers. Proc. ISMB.

[47] Nishimura K (2009). An auxin-based degron system for the rapid depletion of proteins in nonplant cells. Nat. Methods.

