# Supplementary material for: Divalent metal transporter-related protein restricts animals to marine habitats
Source: Commun Biol. 2021 Apr 12;4:463. doi: 10.1038/s42003-021-01984-8 (PMC8041893; doi:10.1038/s42003-021-01984-8)
Supplement: Supplementary file 1 — Supplementary Information [file 42003_2021_1984_MOESM1_ESM.pdf]

Divalent metal transporter-related protein restricts animals to marine habitats

Mieko Sassa [1, 2 *], Toshiyuki Takagi [2], Azusa Kinjo [2], Yuki Yoshioka [1, 2], Yuna Zayasu [3], Chuya Shinzato [2], Shinji Kanda [2], Naoko Murakami-Sugihara [2], Kotaro Shirai [2], Koji Inoue [1, 2]

1 Graduate School of Frontier Sciences, The University of Tokyo, 5-1-5 Kashiwanoha, Kashiwa-shi, Chiba-ken, Japan 277-8561
2 Atmosphere and Ocean Research Institute, The University of Tokyo, 5-1-5, Kashiwanoha, Kashiwa-shi, Chiba, Japan 277-8564
3 Marine Genomics Unit, Okinawa Institute of Science and Technology Graduate University, 1919-1 Tancha, Onna-son, Kunigami-gun, Okinawa, Japan 904-0495

*corresponding author
Mieko Sassa
e-mail address: sassam@aori.u-tokyo.ac.jp

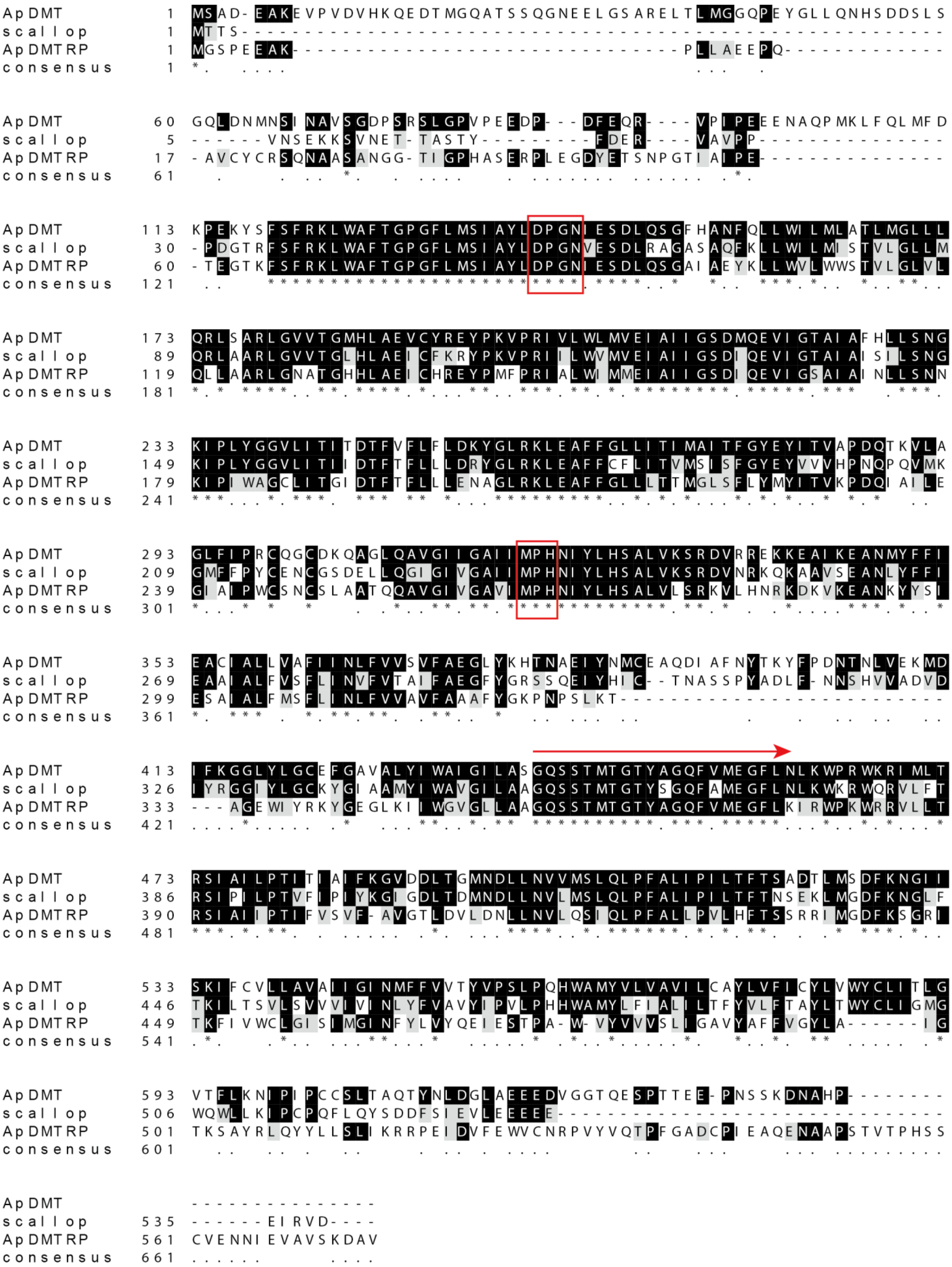

Fig. S1. An alignment result of ApDMT, ApDMTRP and scallop DMT. Alignment was carried out with MAFFT version 7. Black shaded character indicate the identical amino acids with asterisk and gray box indicate not identical but at least similar to the column-consensus with dot. Red arrows indicate consensus transport motifs (CTMs). Red boxes indicate functionally important amino acid residues.

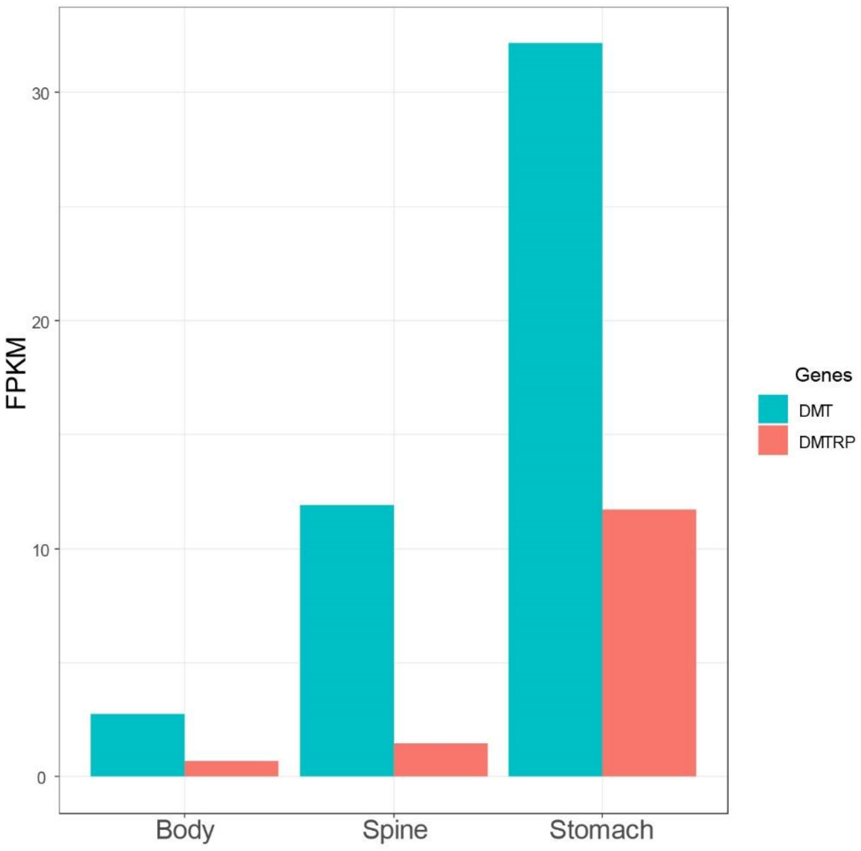

Fig. S2. FPKM data of the crown-of-thorns starfish (COTS), *Acanthaster planci*, divalent metal transporter (ApDMT) and DMT-related protein (ApDMTRP) in COTS body, spine and stomach. Data were obtained from the Marine Genomics Unit Genome Sequencing / Annotation Projects database https://marinegenomics.oist.jp/cots/blast/search?project_id=46.

Table S1. Primers used in this study.

objective	name	sequence
ApDMT cloning	ApDMT-5UTR	AGACTGAGAATTGATTATAGCCTTG
	ApDMT-3UTR	GAGTAATGCTACCCGCCATC
	KozakApDMT	GCCACCATGTCTGCTGATGAAGCCAA
	ApDMT3	CTACGGATGGGCGTTGTCTT
ApDMTRP cloning	ApRP-5UTR	GGAGCGAGATAACCCAGCTA
	ApRP-3UTR	ACACACACGGACAGGGTTTT
	KozakApRP	GCCACCATGGGAAGCCCAGAGGAAGC
	ApRP3	TCACACAGCATCCTTTGACA
Insert check	T7	TAATACGACTCACTATAGGGCG
	SP6	ATTTAGGTGACACTATAGAATACTCAAG
Subcellular localization	pDR+EGFP	TCTCGGCATGGACGAGCTGTACAAGGCGAATTTCTTATGA
	ApDMT+EGFP	GACAACGCCCATCCGATGGTGAGCAAGGGCGAGGAGCTGT
	ApRP+EGFP	TCAAAGGATGCTGTGATGGTGAGCAAGGGCGAGGAGCTGT
Yeast construct	pDR+ApDMT5	TATACCCCAGCCTCGATGTCTGCTGATGAAGCCAA
	pDR+ApDMT3	TCATAAGAAATTCGCCTACGGATGGGCGTTGTCTT
	pDR+ApRP5	TATACCCCAGCCTCGATGGGAAGCCCAGAGGAAGC
	pDR+ApRP3	TCATAAGAAATTCGCTCACACAGCATCCTTTGACA
	ADH5	GCGAATTTCTTATGATTTATGATTT
	pPMA3	CGAGGCTGGGGTATATTTTTTTTCT

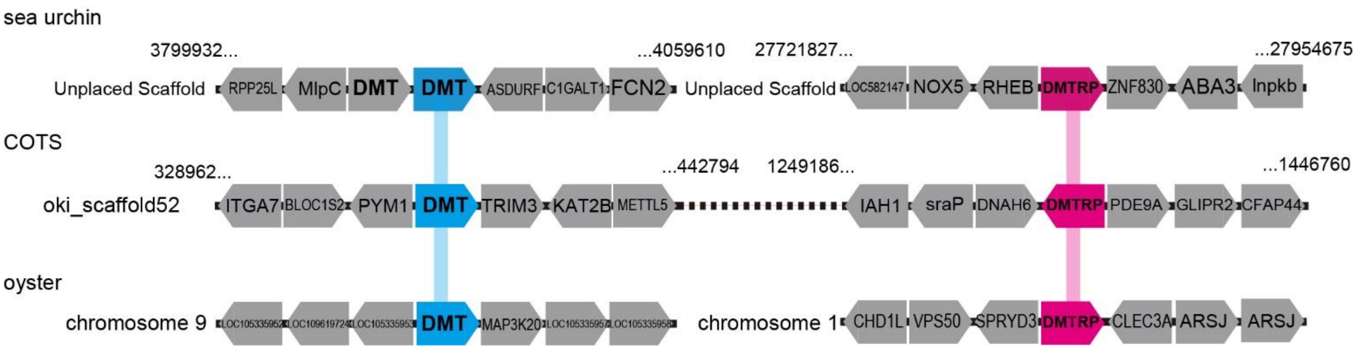

Fig. S3. Genes around divalent metal transporter (DMT) and DMT-related protein (DMTRP) genes in the sea urchin, *Strongylocentrotus purpuratus,* crown-of-thorns starfish (COTS), Acanthaster planci and the oyster *Crassostrea gigas*. Scaffold names and chromosome numbers are indicated on the left. Genes and their orientations are indicated by pentagons of the same size. DMT, DMTRP, and other genes are indicated in blue, magenta, and grey. Numbers above the COTS and sea urchin scaffolds indicate start or end positions of gene locations. Protein names and gene IDs are listed in Table S3.

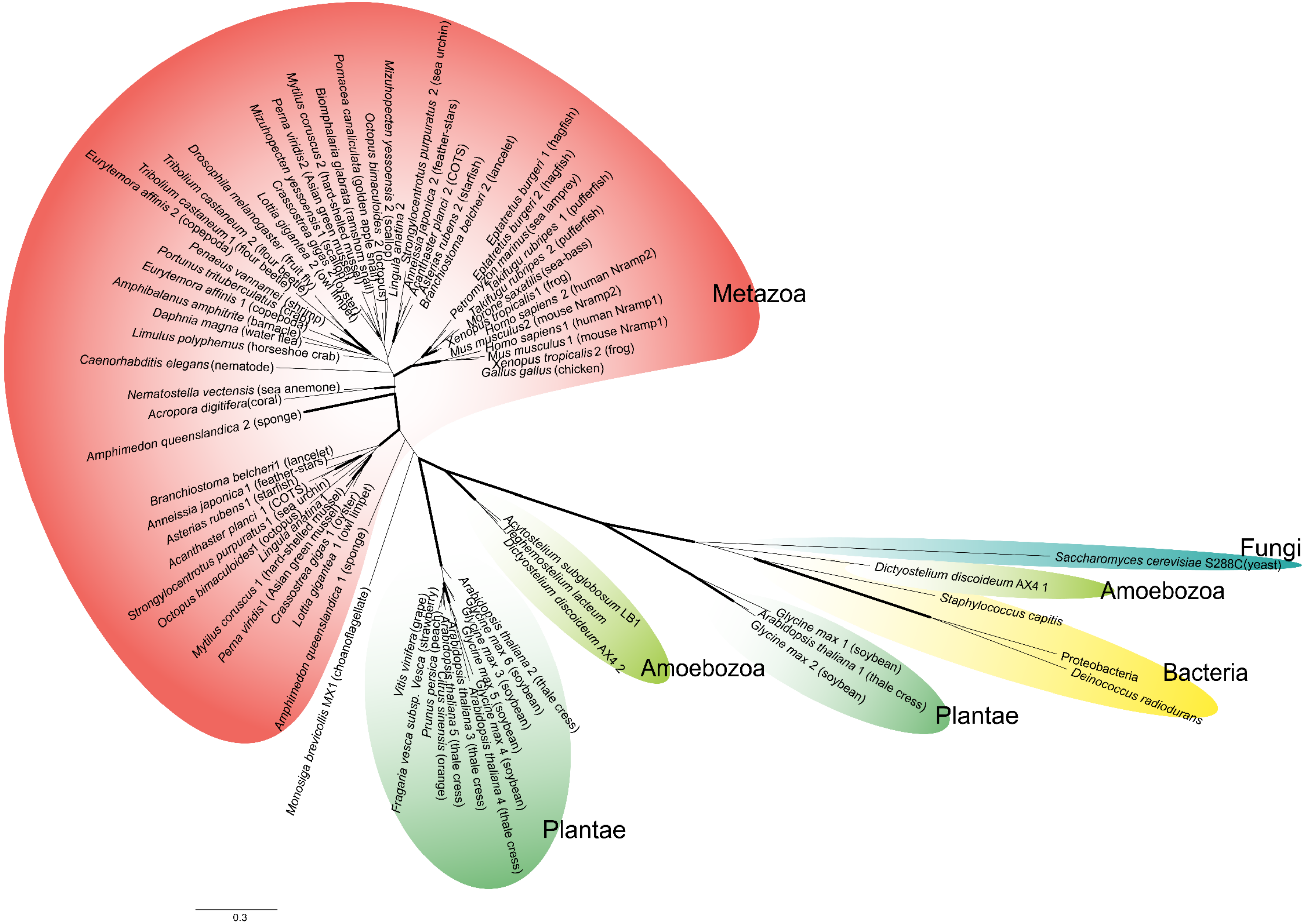

Fig. S4. An unrooted tree constructed using sequences of bacteria, amoebozoans and yeast, plants, and metazoans listed in Table S2. Segments with bootstrap values more than 70% are indicated by bold lines. The scale bar represents a phylogenetic distance of 0.3 substitutions per site.

Table S2. The name and ID of genes in the vicinity of COTS, European starfish, sea urchin and oyster divalent metal transporter (DMT) and DMT-related protein (DMTRP) genes.

	Gene	Protein	gene ID
COTS			
oki_scaffold52	ITGA7	integrin alpha-7-like	110979314
	BLOC1S2	biogenesis of lysosome-related organelles complex 1 subunit 1-like	110979330
	PYM1	partner of Y14 and mago-like	110979329
	DMT	natural resistance-associated macrophage protein 2-like	110979328
	TRIM3	tripartite motif-containing protein 3-like	110979346
	KAT2B	histone acetyltransferase KAT2B-like	110979345
	METTL5	methyltransferase-like protein 5	110979347
oki_scaffold52	CFAP44	cilia- and flagella-associated protein 44-like	110979359
	GLIPR2	Golgi-associated plant pathogenesis-related protein 1-like	110979412
	PDE9A	high affinity cGMP-specific 3',5'-cyclic phosphodiesterase 9A-like	110979413
	DMTRP	metal transporter Nramp3-like	110979403
	DNAH6	dynein heavy chain 6, axonemal-like	110979308
	sraP	serine-rich adhesin for platelets-like	110979440
	IAH1	isoamyl acetate-hydrolyzing esterase 1 homolog	110979310
European starfish			
Chromosome 15	ANKAR	ankyrin and armadillo repeat-containing protein-like	117300372
	OSGEPL1	probable tRNA N6-adenosine threonylcarbamoyltransferase, mitochondrial	117299966
	TRIM23	E3 ubiquitin-protein ligase TRIM23-like	117299967
	DMT	natural resistance-associated macrophage protein 2-like	117300019
	PYM1	partner of Y14 and mago-like	117300020
	BLOC1S2	biogenesis of lysosome-related organelles complex 1 subunit 1-like	117300068
	LOC117300338	uncharacterized LOC117300338	117300338
Chromosome 3	LOC117287777	uncharacterized LOC117287777	117287777
	GABBR2	gamma-aminobutyric acid type B receptor subunit 2-like	117288444

	GABBR2	gamma-aminobutyric acid type B receptor subunit 2-like	117288272
	DMTRP	metal transporter Nramp3-like	117288171
	DNAH6	dynein heavy chain 6, axonemal-like	117288170
	EGF1	fibropellin-1-like	117288040
	EGF1	fibropellin-1-like	117288042
Sea urchin			
Unplaced Scaffold	RPP25L	ribonuclease P protein subunit p25-like protein	593477
	MlpC	myosin-like protein MlpC	373228
	ASDURF	ASNSD1 upstream open reading frame protein-like	115925970
	C1GALT1	glycoprotein-N-acetylgalactosamine 3-beta-galactosyltransferase 1	100888543
	FCN2	ficolin-2	576297
Unplaced Scaffold	LOC582147	LOC582147 uncharacterized LOC582147	582147
	NOX5	NADPH oxidase 5-like	115919487
	RHEB	GTP-binding protein Rheb	581717
	ZNF830	zinc finger protein 830-like	115919307
	ABA3	molybdenum cofactor sulfurase	583644
	lnpkb	endoplasmic reticulum junction formation protein lunapark-B-like	755194
oyster			
Chromosome 9	LOC105335952	endoglucanase-like	105335952
	LOC109619724	endoglucanase	109619724
	LOC105335953	endoglucanase	105335953
	MAP3K20	mitogen-activated protein kinase kinase kinase 20	105335956
	LOC105335957	uncharacterized LOC105335957	105335957
	LOC105335958	uncharacterized LOC105335958	105335958
Chromosome 1	CHD1L	chromodomain-helicase-DNA-binding protein 1-like	105339993
	VPS50	syndetin	105339971
	SPRYD3	SPRY domain-containing protein 3	105339972
	CLEC3A	C-type lectin domain family 3 member A homolog	109620190

	ARSJ	arylsulfatase J	105339974
	ARSJ	arylsulfatase J-like	117690967
