# Supplementary material for: Divalent metal transporter-related protein restricts animals to marine habitats
Source: Commun Biol. 2021 Apr 12;4:463. doi: 10.1038/s42003-021-01984-8 (PMC8041893; doi:10.1038/s42003-021-01984-8)
Supplement: Supplementary file 2 — Description of Additional Supplementary Files [file 42003_2021_1984_MOESM2_ESM.pdf]

Description of Additional Supplementary Files

File name: Supplementary Data 1

Description: Divalent metal transporter (DMT)-like sequences used for phylogenetical analysis.

File name: Supplementary Data 2

Description: Source data of Fig. 6 (yeast experiments).
